# The Neglected Intrinsic Resistome of Bacterial Pathogens

**DOI:** 10.1371/journal.pone.0001619

**Published:** 2008-02-20

**Authors:** Alicia Fajardo, Nadia Martínez-Martín, María Mercadillo, Juan C. Galán, Bart Ghysels, Sandra Matthijs, Pierre Cornelis, Lutz Wiehlmann, Burkhard Tümmler, Fernando Baquero, José L. Martínez

**Affiliations:** 1 Departamento de Biotecnología Microbiana, Centro Nacional de Biotecnología, Consejo Superior de Investigaciones Científicas (CSIC), Cantoblanco, Madrid, Spain; 2 Unidad Asociada al Centro Nacional de Biotecnología, Consejo Superior de Investigaciones Científicas (CSIC) “Resistencia a los antibióticos y virulencia bacteriana”, Hospital Ramón y Cajal, Madrid, Spain; 3 CIBER Epidemiología y Salud Pública (CIBERESP), Hospital Ramón y Cajal, Madrid, Spain; 4 Departamento de Microbiología, Hospital Ramón y Cajal, Madrid, Spain; 5 Laboratory of Microbial Interactions, Department of Molecular and Cellular Interactions, Flanders Interuniversity Institute for Biotechnology, Vrije Universiteit Brussel, Brussels, Belgium; 6 Klinische Forschergruppe, Medizinische Hochschule Hannover, Hannover, Germany; Alfa Institute of Biomedical Sciences (AIBS), Greece

## Abstract

Bacteria with intrinsic resistance to antibiotics are a worrisome health problem. It is widely believed that intrinsic antibiotic resistance of bacterial pathogens is mainly the consequence of cellular impermeability and activity of efflux pumps. However, the analysis of transposon-tagged *Pseudomonas aeruginosa* mutants presented in this article shows that this phenotype emerges from the action of numerous proteins from all functional categories. Mutations in some genes make *P. aeruginosa* more susceptible to antibiotics and thereby represent new targets. Mutations in other genes make *P. aeruginosa* more resistant and therefore define novel mechanisms for mutation-driven acquisition of antibiotic resistance, opening a new research field based in the prediction of resistance before it emerges in clinical environments. Antibiotics are not just weapons against bacterial competitors, but also natural signalling molecules. Our results demonstrate that antibiotic resistance genes are not merely protective shields and offer a more comprehensive view of the role of antibiotic resistance genes in the clinic and in nature.

## Introduction

The large increase in human life expectancy during the last century is attributable, among other factors, to the effectiveness of antibiotics in curing formerly devastating infectious diseases and, particularly in enabling the application of effective but aggressive medical and surgical techniques to elderly, weak and immunodepressed patients. For this reason, the increase in antibiotic resistance of human pathogens [1], [2] affects not only the treatment of infectious diseases, but also many other medical practices, such as immunosuppression in transplants, anticancer chemotherapy, and advanced surgery. As stated by the World Health Organization, the spread of antibiotic-resistant bacteria at hospitals “…means that commonplace medical procedures once previously taken for granted could be conceivably consigned to medical limbo. The repercussions are almost unimaginable” [3]. From this perspective, studies addressing possible means to minimize and eventually predict antibiotic resistance [4] must be considered a priority for the future health of humankind.

To understand and predict the future evolution of antibiotic resistance in bacterial pathogens, two different problems need to be addressed: acquisition [5] of resistance genes (or mutations [6]) by formerly susceptible bacteria, and intrinsic antibiotic resistance. The existence of previously unknown determinants of antibiotic resistance that could be transferred to human pathogens and might eventually constitute a health problem was addressed in a recent report [7] in which it was shown that microbial antibiotic producers in the environment have developed a large and versatile arsenal of antibiotic resistance genes (resistome [8]).

In the current study, we focused on the second problem, the existence of species-specific bacterial intrinsic resistome, defined as the ensemble of chromosomal genes that are involved in intrinsic resistance and whose presence in strains of a bacterial species is independent of previous antibiotic exposure and is not due horizontal gene transfer (HGT). Noteworthy, the most prominent intrinsically-resistant bacteria have an environmental (non-clinical) origin, in habitats that are much less likely than clinical settings to present intense antibiotic selective pressure. This suggests [9] that the main physiological role of the elements involved in this phenotype in the natural habitats of these bacteria in not conferring resistance to antibiotics currently in use in clinical practice. The intrinsic resistome is thus an evolutionarily ancient phenotype common to all strains of a bacterial species that has not been acquired as a result of recent utilization of antibiotics for therapy.

To address the elements involved in the building-up of bacterial intrinsic resistance, we investigated the intrinsic resistome of the model organism *Pseudomonas aeruginosa*. [10], [11], [12], [13]. This bacterial species is the most important gram-negative nosocomial pathogen with an environmental origin [13]. *P. aeruginosa* produces infections, not only at hospitals [14], but associated to other basal pathologies such as cystic fibrosis or HIV [10], [15], [16]. Besides its virulence, one important feature of *P. aeruginosa* consists on its characteristic low susceptibility to antibiotics [17], [18], [19]. It is worth to state that *P. aeruginosa* is a free-living organisms capable to thrive in different environments [20]. Furthermore, it has been shown that this bacterial species utilizes the same virulence determinants to infect different hosts from plants to humans [21], [22], [23], [24], [25], and that infective and environmental strains are equivalent at the genomic [26], [27] and physiological levels [28]. All these indicate that several phenotypes relevant for *P. aeruginosa* infection, including its characteristic low susceptibility to antibiotics, have been selected in natural environments distant from human contact.

Intrinsic resistance to antibiotics is thought to result from the reduced permeability of the bacterial envelope and the activity of multidrug efflux pumps [29]. However, this lacks experimental proof because an exhaustive search of other types of genes involved in intrinsic resistance has not been performed. Establishment of the genetic networks involved in the antibiotic resistance of bacterial pathogens is thus of paramount relevance to minimizing and predicting resistance, a task that has been recently theoretically addressed [4]. In the present work, and using this theoretical framework, a high-throughput analysis of the elements involved in the intrinsic resistance of a bacterial pathogen is made. The obtained data are important for predicting and fighting resistance and for understanding the functional and ecological role of antibiotic resistance determinants in natural ecosystems.

## Results and Discussion

**Figure 1:**
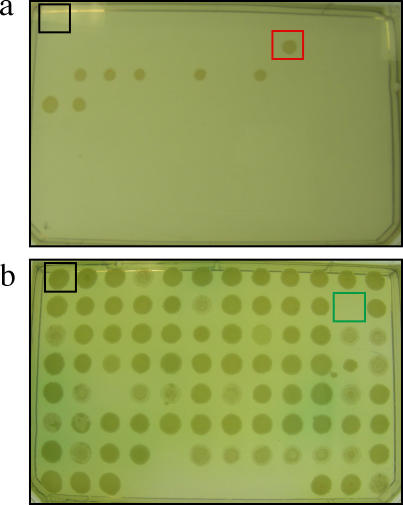
Screening of *P. aeruginosa* mutants with altered antibiotic susceptibility. Two libraries of transposon-tagged *P. aeruginosa* mutants were screened to detect changes in their susceptibility to the antimicrobial agents polymixin B, amikacin, ciprofloxacin, tetracycline, imipenem, and ceftazidime. A mutant was considered resistant (red square in A) if it was able to grow at antibiotic concentrations that inhibited the growth of the wild-type strain (black square in both panels). A mutant was considered hypersusceptible (green square in B) if it was not able to grow at antibiotic concentrations permissive for the wild-type strain.

**Figure 2:**
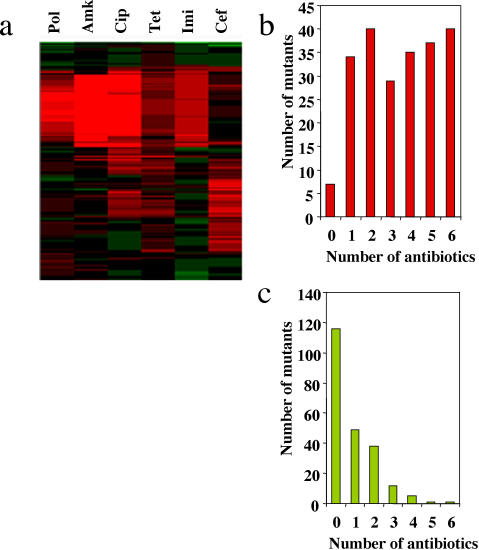
Antibiotic susceptibility of *P. aeruginosa* mutants. The susceptibility of the selected mutants was determined by comparison to the wild-type parental strains. (A) The antibiotic susceptibility ratios of each mutant and its isogenic wild-type strain are shown. The ratios of changes were hierarchically clustered [48] using freely available software (http://rana.lbl.gov/EisenSoftware.htm). Green, more susceptible; red, more resistant; Pol, polymixin B; Amk, amikacin; Cip, ciprofloxacin; Tet, tetracycline; Imi, imipenem; Cef, ceftazidime. Note that in most cases, susceptibilities to several antibiotics changed simultaneously. (B) The number of mutants with higher resistance (resistant mutants) to a given number of tested antibiotics. Most of the mutants had increased resistance to several antibiotics belonging to different structural families. (C) The number of mutants with higher susceptibility (hypersusceptible mutants) to a given number of tested antibiotics. Note that a mutant can be more resistant to some antibiotics (see A) and more susceptible to others and therefore can be included in both (B) and (C).

Two different transposon-tagged insertion libraries of *P. aeruginosa* [30], [31] were screened (Fig. 1) as described in Methods to identify mutants with altered susceptibility to the following 6 antimicrobial agents belonging to different structural families: polymixin B (polymixins), amikacin (aminocyclitols), ciprofloxacin (fluoroquinolones), tetracycline (tetracyclines), imipenem (carbapenems) and ceftazidime (cephalosporins). Altered susceptibility to one or more antibiotics was observed in 222 of the 5952 tested mutants (3.7%, Fig. 2). The position of the transposon was determined by inverse-polymerase chain reaction [31], sequencing of the amplicons, and comparison to the available sequence of *P. aeruginosa* (http://www.pseudomonas.com/). After filtering insertions occurring in the same gene, we have detected 112 loci in the genome of *P. aeruginosa* that contribute to its characteristic phenotype of antibiotic susceptibility (Supporting Online Material, Table S1). Ninety nine of the insertions were located in open reading frames already sequenced in the wild-type strain *P. aeruginosa* PAO1, 8 were located in intergenic regions and 5 were strain specific. Since the genome of *P. aeruginosa* contains 5570 genes, our results indicate that at least 1.8% of the genome of this intrinsically resistant opportunistic pathogen contributes to its characteristic phenotype of susceptibility to antibiotics. Among these 112 loci, only one (a component of a multidrug efflux pump *mexD*) has been previously annotated as an antibiotic resistance gene. Although a few others, such as the putative RND efflux transporters PA1436 or PA2527 are predicted to play a role in *P. aeruginosa* resistance, the large majority of genes emerging form our screen were not previously thought to play any role in the antibiotic susceptibility of this bacterial species. Thus, our work unveils a hidden intrinsic resistome in *P. aeruginosa*.

The combinations of classical antibiotics with drugs that increase bacterial susceptibility, like for example beta-lactams/beta-lactamases inhibitors [32], constitute a valuable therapeutic tool. In the present work, we have identified genes (Supporting Online Material, Table S1) which inactivation increases *P. aeruginosa* susceptibility to several antimicrobials. Those genes define novel targets for the development of anti-resistance drugs such those above described.

Noteworthy, most mutants had altered susceptibility to multiple antibiotics, indicating that the development of resistance is not specific to a given antibiotic. The view that intrinsic resistance has a large degree of non-specificity is also supported by the fact that many of the products encoded by these genes are involved in basic functions of the physiology of *P. aeruginosa* (Table 1). This absence of specificity challenges the idea that each antibiotic resistance gene originates as a means to avoid the activity of a particular antibiotic [33]. Together with the notion that antibiotics do not only function as weapons against competitors in Nature, but also as signal molecules [34], [35], our results demonstrate that antibiotic resistance genes are more than shields, offering a more comprehensive view of their physiological role in bacterial pathogens.

**Table 1:** Functional categories of genes involved in the antibiotic susceptibility of *P. aeruginosa*

Category*	Number of genes in the genome of *P. aeruginosa* PAO1**	Mutants with changes in antibiotic susceptibility belonging to this functional category**
Adaptation, protection	170	2 (1.2%)
Amino acid biosynthesis and metabolism	226	4 (1.8%)
Antibiotic resistance and susceptibility	31	1 (3.3%)
Motility and Attachment	112	4 (3.6%)
Biosynthesis of cofactors, prosthetic groups and carriers	159	2 (1.3%)
Carbon compound catabolism	173	7 (4.05%)
Cell division	29	0 (-)
Cell wall/LPS/capsule	136	1 (0.7%)
Central intermediary metabolism	99	2 (2.0%)
Chaperons and heat shock proteins	54	0 (-)
Chemotaxis	64	1 (1.6%)
DNA replication, recombination, modification and repair	88	1 (1.1%)
Energy metabolism	207	3 (1.4%)
Fatty acid and phospholipid metabolism	62	1 (1.6%)
Hypothetical, unclassified, unknown	2364	31 (1.3%)
Membrane proteins	676	17 (2.5%)
Nucleotide biosynthesis and metabolism	81	1 (1.2%)
Protein secretion/export apparatus	99	2 (2.02%)
Putative enzymes	477	11 (2.3%)
Related to phages, transposons, or plasmids	65	2 (3.1%)
Transcription, RNA processing and degradation: genes	55	0 (-)
Transcriptional regulators	473	5 (0.08)
Translation, post-translational modification, degradation	195	1 (0.5%)
Transport of small molecules	595	17 (2.9%)
Two-component regulatory systems	121	4 (3.3%)
Secreted Factors (toxins, enzymes, alginate): genes	88	1 (1.1%)
Non-coding RNA gene	97	0 (-)
Intergenic region	-	8 (-)
Genes from non-PAO1 strain	-	5 (-)

* Functional categories as defined in http://www.pseudomonas.com/

** Some genes belong to more than one category.

Most mutants exhibited enhanced resistance to antibiotics (Fig. 2B) and 18% of them were resistant to all tested antibiotics. The proteins involved include known and hypothetical outer membrane proteins and transporters such as OmpA, CzcC, PA0809, PA2760 and PA4691. These proteins likely serve to transport the antibiotics inside the cell and thus might represent mechanisms of resistance due to drug impermeability.

However, other mutations reducing susceptibility to all tested antibiotics targeted genes encoding proteins involved in basic cellular metabolism such as *rafDI,* which encodes for ADP-L-glycero-D-mannoheptose-6-epimerase, and *gapA*, which encodes glyceraldehyde 3-phosphate dehydrogenase. Slow-growing bacteria are usually less susceptible to antibiotics [36]. Since strong impairment of growth in rich medium was not observed for any mutant, growth-independent features of the bacterial metabolome must be responsible for the observed changes in antimicrobial susceptibility. This highlights the fact that antibiotic susceptibility is likely dependent on the metabolic state of bacteria [37], [38]. That is, in addition to the well-known specific mechanisms of resistance, bacteria have a “physiology-dependent” resistome. During infection, bacteria sense their environment and alter their metabolism accordingly. Thus, knowledge on the changes that occur in bacterial metabolic networks during infection may help us to improve treatments, mainly at locations in the human-body where the amount of free antibiotic is not very high. The fact that mutations both in intergenic regions and mutations in regulatory proteins and sensor systems affected bacterial susceptibility to antibiotics (see Table 1) also supports the concept that *in vivo* susceptibility [39] might be dependent on the tuning of physiological circuits in response to the inputs received during infection.

Antibiotics have been sought and customized in order to combat bacterial pathogens, and it was thought that their ecological function is to fight competitors in natural (non-clinical) environments. According to this warfare-based view, resistant organisms should develop specific shields (antibiotic resistance genes) to avoid the action of antibiotics. The acquisition of antibiotic resistance genes by HGT supports this view, since such genes use to be specific to a given structural family of antibiotics and, in a few cases, the same gene is present in the producer organism and in the bacterial pathogen [40]. Nevertheless, we must remember that after HGT, classical antibiotic resistance genes are displaced from the genetic background in which they evolved and their function in the original organism is not necessarily antibiotic resistance. Examples are *P. aeruginosa* strains that were isolated from non clinical environments before the discovery of quinolones but that have active mechanisms of resistance to these synthetic drugs [28], and some antibiotic-inactivating enzymes, prototypes of canonical antibiotic resistance genes, that exert functions unrelated to antibiotics and important to bacterial physiology [41], [42]. These mechanisms of resistance were not selected in the original organism to avoid the action of an antibiotic, although they can provide this advantage upon transfer to a new host, in what has been named an exaptation evolutionary process [43]. As stated above, our results provide novel evidences supporting the notion that some determinants involved in basic bacterial physiology in natural habitats may have an important role in resistance to environments with a high load of antibiotics, such as hospital settings.

Along our work, we found that the development of an intrinsic resistance phenotype in *P. aeruginosa* requires the concerted action of several genes that encode basic functions of cell physiology. Our results support the notion that intrinsic resistance, and the capacity to acquire higher levels of resistance may be the consequence of complex networks of interactions among several bacterial proteins; this opens a new field of research with regard to predicting antibiotic resistance [4] in bacterial populations.

Several of the selected mutants are more resistant to antibiotics than is the wild-type strain, such that mutations in these genes might eventually play a role in acquired resistance during treatment. The mechanisms of mutation-driven resistance are usually purposefully sought using genes for which a role in antibiotic resistance has already been described. Conversely, as yet unknown antibiotic resistance genes, such as those described here, have been ignored when examining the molecular basis of mutation-driven resistance in *P. aeruginosa*.

In conclusion, intrinsic resistance to antibiotics and the ability to evolve to higher levels of resistance involve a complex network of elements, including non-classical antibiotic-resistance genes. Characterization of the genes forming these networks will contribute to accurate prediction of the emergence and evolution of antibiotic resistance [4] and help to define new targets (hypersusceptible mutants) for identifying antimicrobials against intrinsically resistant bacterial pathogens.

Our work supports the notion that antibiotic resistance elements are not just shields that evolved as protection mechanisms against antibiotic weapons, but have several other functions relevant for bacterial physiology. This is a Copernican turning point in our view concerning antibiotic resistance and the functional and ecological role of these elements in hospitals and in non-clinical environments.

## Materials and Methods

### Bacterial strains and growth conditions

Two libraries of transposon-tagged *P. aeruginosa* mutants were used. One of the libraries comprised 3936 mutants and has been obtained from the *P. aeruginosa* Tb isolate [30]. The other contained 2016 mutants and has been obtained from the *P. aeruginosa* 59.20 strain [31]. For all experiments concerning antibiotic susceptibility, bacteria were grown at 37°C in Mueller-Hinton medium [44]. For obtaining chromosomal DNA, bacteria were grown overnight at 37°C in LB broth without glucose [45] in a rotary shaker operated at 220 rpm.

### Screening of the libraries and determination of Minimal Inhibitory Concentrations (MICs)

The screening was performed using Mueller-Hinton [44] agar plates containing increasing concentrations (2-fold each plate) of the following antimicrobial agents: polymixin B, amikacin, ciprofloxacin, tetracycline, imipenem, ceftazidime. In all cases, 5 µl drops, each one containing around 10^4^ colony-forming-units of each mutant, were poured onto the plates, and the growth of the spots was recorded after 24 h of growth. For comparison purposes, all plates contained a spot with the corresponding wild-type strain. In all cases, the tests were replicated at least three times. A mutant was considered resistant if was capable to grow at antibiotic concentrations that inhibited the growth of its parental, wild-type strain. A mutant was classified as hypersusceptible to a given antibiotic if did not grow at concentrations in which its parental wild-type strain was capable to grow.

For all selected mutants, MICs to the six antibiotics were determined in Mueller-Hinton [44] agar plates as described [46].

### Determination of the point of insertion of the transposon and identification of the mutated gene

Chromosomal DNA was obtained from the selected mutants using the GENOME DNA KIT (MP Biomedicals). For each mutant, 1 µg of genomic DNA was cut using 20 units of the restriction enzyme PstI (Biolabs Inc.). The reaction was performed at 37°C overnight. After inactivation of the restriction enzyme at 80°C for 20 minutes, 100 ng of the digested DNA was ligated with 200 units of T4 DNA ligase at 16°C overnight in 200 µl of ligation buffer (50 mM Tris-HCl, 10 mM MgCl_2_, 10 mM DTT, 1 mM ATP, 25 µg/ml BSA, pH 7,5). These conditions favor autoligation and circularization of the fragments. After ligation, DNA was precipitated by adding 20 µl of 3 M sodium acetate, pH 5.2 and 500 µl of ethanol 100%. The mixture was incubated one hour at –20°C, and the DNA was pelleted by centrifugation. Pelleted DNA was washed with ethanol 70% and, after further centrifugation, dried at 37°C. This dried DNA was used for inverse-PCR of the circularized fragments generated from the chromosomal DNA obtained from each mutant.

Inverse-PCR was made using the primers described in Table 2. Primers Gm1 and phoA5 were used to map the location of the transposon miniTnphoA3 in mutants belonging to the library made in the strain 59.20 and primers Tn5c and Tn5d were used to map the location of the transposon miniTn5 in the mutants belonging to the library made in the strain TB. In all cases, the PCR reaction was made in 50 µl reaction mixture containing 5 µl of PCR buffer 10X (27.5 mM MgCl_2_, 20 mM Tris-HCl, 100 mM KCl, 1 mM DTT, 0.1 mM EDTA, 0.5% Nonidet P40 v/v, 0.5% Tween v/v, 50% glycerol v/v) each deoxynucleotide (dCTP, dGTP. dATP and dTTP) at a concentration of 350 µM each, 300 nM of each primer, 2.5 units of Expand Long Template Enzyme mix (Roche) and the template DNA obtained as described above. The mixture was heated at 94°C for 2 min, followed by 30 cycles of 10 s at 94°C, 30 s at 55°C and 6 min at 68°C, and finally one 7 min extension step at 68°C before the end of the reaction. PCR products were run on 1% agarose gels and stained with ethidium bromide [47]. For some mutants an amplicon was not obtained using these experimental conditions. In these cases, the experiment was repeated using the each one of the restriction enzymes BstEII, NarI, SfoI, BamHI instead of PstI for the first DNA digestion step. Before sequencing, the amplicons were purified using QIAquick PCR purification protocol (Quiagen).

**Table 2:** Primers used for determining the location of the insertions

Primer name	5′-3′ Sequence	Transposon
Gm1	TGGACCAGTTGCGTGAGCGCAT	mini TnphoA3
phoA5	GCGGCAGTCTGATCACCCGTTA	mini TnphoA3
Tn5c	TGCTGGCCTTTTGCTCACAT	mini Tn5
Tn5d	CGAACCGAACAGGCTTATGTC	mini Tn5

After purification, the amplicons were sequenced by the dideoxi termination method with the same primers used for the PCR reaction. The obtained sequences were used to make a Blast search of the *P. aeruginosa* PAO1 genome (www.pseudomonas.com) in order to map the position of the inserted transposon. In some few mutants, the sequences were not present in the chromosome of *P. aeruginosa* PAO1, and a general Blast search was performed. For every mutant, the two sequences obtained with both primers matched in the same gene or intergenic region of the sequenced chromosome. This assures the reliability of the method used for mapping the insertions.

## Supporting Information

Table S1 Relative susceptibility and chromosomal position of *P. aeruginosa* mutations that alter the level of antibiotic susceptibility.
(0.16 MB DOC)

## References

[1] Levy SB (1998). Multidrug resistance–a sign of the times.. N Engl J Med.

[2] Smith DL, Dushoff J, Perencevich EN, Harris AD, Levin SA (2004). Persistent colonization and the spread of antibiotic resistance in nosocomial pathogens: resistance is a regional problem.. Proc Natl Acad Sci U S A.

[3] World HO (2000). Overcoming antibiotic resistance..

[4] Martinez JL, Baquero F, Andersson DI (2007). Predicting antibiotic resistance.. Nat Rev Microbiol.

[5] Davies JE (1997). Origins, acquisition and dissemination of antibiotic resistance determinants.. Ciba Found Symp.

[6] Martinez JL, Baquero F (2000). Mutation frequencies and antibiotic resistance.. Antimicrob Agents Chemother.

[7] D'Acosta VM, McGrann KM, Hughes DW, Wright GD (2006). Sampling the antibiotic resistome.. Science.

[8] Wright GD (2007). The antibiotic resistome: the nexus of chemical and genetic diversity.. Nat Rev Microbiol.

[9] Alonso A, Sanchez P, Martinez JL (2001). Environmental selection of antibiotic resistance genes.. Environ Microbiol.

[10] Quinn JP (1998). Clinical problems posed by multiresistant nonfermenting gram-negative pathogens.. Clin Infect Dis.

[11] Stover CK, Pham XQ, Erwin AL, Mizoguchi SD, Warrener P (2000). Complete genome sequence of Pseudomonas aeruginosa PA01, an opportunistic pathogen.. Nature.

[12] Gales AC, Jones RN, Turnidge J, Rennie R, Ramphal R (2001). Characterization of Pseudomonas aeruginosa isolates: occurrence rates, antimicrobial susceptibility patterns, and molecular typing in the global SENTRY Antimicrobial Surveillance Program, 1997–1999.. Clin Infect Dis.

[13] Navon-Venezia S, Ben-Ami R, Carmeli Y (2005). Update on Pseudomonas aeruginosa and Acinetobacter baumannii infections in the healthcare setting.. Curr Opin Infect Dis.

[14] Vincent JL, Bihari DJ, Suter PM, Bruining HA, White J (1995). The prevalence of nosocomial infection in intensive care units in Europe. Results of the European Prevalence of Infection in Intensive Care (EPIC) Study. EPIC International Advisory Committee.. Jama.

[15] Govan JR, Deretic V (1996). Microbial pathogenesis in cystic fibrosis: mucoid Pseudomonas aeruginosa and Burkholderia cepacia.. Microbiol Rev.

[16] Gibson RL, Burns JL, Ramsey BW (2003). Pathophysiology and management of pulmonary infections in cystic fibrosis.. Am J Respir Crit Care Med.

[17] Hancock RE (1998). Resistance mechanisms in Pseudomonas aeruginosa and other nonfermentative gram-negative bacteria.. Clin Infect Dis.

[18] Hancock RE, Speert DP (2000). Antibiotic resistance in Pseudomonas aeruginosa: mechanisms and impact on treatment.. Drug Resist Updat.

[19] Gatell JM, Marrades R, el-Ebiary M, Torres A (1996). Severe pulmonary infections in AIDS patients.. Semin Respir Infect.

[20] Spiers AJ, Buckling A, Rainey PB (2000). The causes of Pseudomonas diversity.. Microbiology.

[21] Rahme LG, Stevens EJ, Wolfort SF, Shao J, Tompkins RG (1995). Common virulence factors for bacterial pathogenicity in plants and animals.. Science.

[22] Mahajan-Miklos S, Tan MW, Rahme LG, Ausubel FM (1999). Molecular mechanisms of bacterial virulence elucidated using a Pseudomonas aeruginosa-Caenorhabditis elegans pathogenesis model.. Cell.

[23] Tan MW, Mahajan-Miklos S, Ausubel FM (1999). Killing of Caenorhabditis elegans by Pseudomonas aeruginosa used to model mammalian bacterial pathogenesis.. Proc Natl Acad Sci U S A.

[24] Sifri CD, Begun J, Ausubel FM (2005). The worm has turned–microbial virulence modeled in Caenorhabditis elegans.. Trends Microbiol.

[25] Navas A, Cobas G, Talavera M, Ayala JA, Lopez JA (2007). Experimental validation of Haldane's hypothesis on the role of infection as an evolutionary force for Metazoans.. Proc Natl Acad Sci U S A.

[26] Morales G, Wiehlmann L, Gudowius P, van Delden C, Tummler B (2004). Structure of Pseudomonas aeruginosa populations analyzed by single nucleotide polymorphism and pulsed-field gel electrophoresis genotyping.. J Bacteriol.

[27] Wiehlmann L, Wagner G, Cramer N, Siebert B, Gudowius P (2007). Population structure of Pseudomonas aeruginosa.. Proc Natl Acad Sci U S A.

[28] Alonso A, Rojo F, Martinez JL (1999). Environmental and clinical isolates of Pseudomonas aeruginosa show pathogenic and biodegradative properties irrespective of their origin.. Environ Microbiol.

[29] Nikaido H (2001). Preventing drug access to targets: cell surface permeability barriers and active efflux in bacteria.. Semin Cell Dev Biol.

[30] Wiehlmann L, Salunkhe P, Larbig K, Ritzka M, Tummler B (2002). Signature tagged mutagenesis of *Pseudomonas aeruginosa*.. Genome Letters.

[31] de Chial M, Ghysels B, Beatson SA, Geoffroy V, Meyer JM (2003). Identification of type II and type III pyoverdine receptors from Pseudomonas aeruginosa.. Microbiology.

[32] Lee N, Yuen KY, Kumana CR (2003). Clinical role of beta-lactam/beta-lactamase inhibitor combinations.. Drugs.

[33] Benveniste R, Davies J (1973). Aminoglycoside antibiotic-inactivating enzymes in actinomycetes similar to those present in clinical isolates of antibiotic-resistant bacteria.. Proc Natl Acad Sci U S A.

[34] Linares JF, Gustafsson I, Baquero F, Martinez JL (2006). Antibiotics as intermicrobial signaling agents instead of weapons.. Proc Natl Acad Sci U S A.

[35] Yim G, Wang HH, Davies J (2007). Antibiotics as signalling molecules.. Philos Trans R Soc Lond B Biol Sci.

[36] Levin BR, Rozen DE (2006). Non-inherited antibiotic resistance.. Nat Rev Microbiol.

[37] Wiuff C, Zappala RM, Regoes RR, Garner KN, Baquero F (2005). Phenotypic tolerance: antibiotic enrichment of noninherited resistance in bacterial populations.. Antimicrob Agents Chemother.

[38] Hogan D, Kolter R (2002). Why are bacteria refractory to antimicrobials?. Curr Opin Microbiol.

[39] Martinez JL, Blazquez J, Baquero F (1994). Non-canonical mechanisms of antibiotic resistance.. Eur J Clin Microbiol Infect Dis.

[40] Pang Y, Brown BA, Steingrube VA, Wallace RJ, Roberts MC (1994). Tetracycline resistance determinants in Mycobacterium and Streptomyces species.. Antimicrob Agents Chemother.

[41] Macinga DR, Rather PN (1999). The chromosomal 2′-N-acetyltransferase of Providencia stuartii: physiological functions and genetic regulation.. Front Biosci.

[42] Ainsa JA, Perez E, Pelicic V, Berthet FX, Gicquel B (1997). Aminoglycoside 2′-N-acetyltransferase genes are universally present in mycobacteria: characterization of the aac(2′)-Ic gene from Mycobacterium tuberculosis and the aac(2′)-Id gene from Mycobacterium smegmatis.. Mol Microbiol.

[43] Gould SJ, Lloyd EA (1999). Individuality and adaptation across levels of selection: how shall we name and generalize the unit of Darwinism?. Proc Natl Acad Sci U S A.

[44] Atlas RM (1993). Handbook of Microbiological Media; Parks LC, editor..

[45] Luria SE, Burrous JW (1957). Hybridization between Escherichia coli and Shigella.. J Bacteriol.

[46] Alonso A, Campanario E, Martinez JL (1999). Emergence of multidrug-resistant mutants is increased under antibiotic selective pressure in Pseudomonas aeruginosa.. Microbiology.

[47] Sambrook J, Russell DW (2001). Molecular Cloning. A laboratory manual 3rd. edition..

[48] Eisen MB, Spellman PT, Brown PO, Botstein D (1998). Cluster analysis and display of genome-wide expression patterns.. Proc Natl Acad Sci U S A.

