# Supplementary material for: The Neglected Intrinsic Resistome of Bacterial Pathogens
Source: PLoS One. 2008 Feb 20;3(2):e1619. doi: 10.1371/journal.pone.0001619 (PMC2238818; doi:10.1371/journal.pone.0001619)
Supplement: Table S1 — Relative susceptibility and chromosomal position of P. aeruginosa mutations that alter the level of antibiotic susceptibility. (0.16 MB DOC) [file pone.0001619.s001.doc]

**Table S1. Relative susceptibility and chromosomal position of *P. aeruginosa* mutations that alter the level of antibiotic susceptibility.**

| **PA number** | Gene name and function of the encoded protein as defined in http://www.pseudomonas.com/ | **Relative antibiotic susceptibility*** | | | | | |
| --- | --- | --- | --- | --- | --- | --- | --- |
|  |  | **Pol** | **Amk** | **Cip** | **Tet** | **Imi** | **Cef** |
| 0049 | hypothetical protein | -1 | 0 | -1 | 1 | 0 | 2 |
| 0071 | hypothetical protein | 2 | 0 | 0 | 0 | 1 | 1 |
| 0290 | hypothetical protein | 1 | 0 | 3 | 2 | -1 | 1 |
| 0498 | hypothetical protein | 5 | 6 | 6 | 3 | 4 | 0 |
| 0599 | hypothetical protein | 4 | 6 | 6 | 2 | 6 | 0 |
| 0690 | hypothetical protein | 5 | 6 | 6 | 3 | 4 | 0 |
| 0788 | hypothetical protein | 1 | 1 | 3 | 3 | 0 | 3 |
| 0856 | hypothetical protein | 0 | 0 | -1 | 1 | -1 | -1 |
| 1031 | conserved hypothetical protein. COG predictions: Uncharacterized protein conserved in bacteria [Function unknown]. | 3 | 6 | 6 | 3 | 4 | 1 |
| 1076 | hypothetical protein | 1 | 0 | -1 | 2 | 0 | 4 |
| 1408** | hypothetical protein. COG predictions: Small-conductance mechanosensitive channel [Cell envelope biogenesis, outer membrane]. | 1 | 0 | 3 | 3 | 0 | 5 |
| 3329 | hypothetical protein | -1 | 1 | -1 | 1 | -1 | 0 |
| 3472 | hypothetical protein. COG predicitions: Spr, Cell wall-associated hydrolases (invasion-associated proteins) [Cell envelope biogenesis, outer membrane]. | 1 | 0 | 2 | 2 | 1 | 3 |
| 3728 | hypothetical protein. COG predictions: Smc, Chromosome segregation ATPases [Cell division and chromosome partitioning]. | 1 | 0 | 0 | 1 | 0 | 2 |
| 3731 | conserved hypothetical protein. Alternate gene name: *yjfJ.* COG predictions: PspA, Phage shock protein A (IM30), suppresses sigma54-dependent transcription [Transcription / Signal transduction mechanisms]. | 6 | 6 | 4 | 2 | 4 | 0 |
| 3756 | yafK. hypothetical protein. Uncharacterized protein conserved in bacteria [Function unknown]. | -1 | 0 | 3 | 1 | -1 | -1 |
| 3893** | conserved hypothetical protein COG predictions: Predicted membrane protein [Function unknown]. | 0 | 0 | -1 | 1 | 0 | 3 |
| 4163 | hypothetical protein. COG predictions: GatA, Asp-tRNAAsn/Glu-tRNAGln amidotransferase A subunit and related amidases [Translation, ribosomal structure and biogenesis]. | 0 | 2 | 2 | 2 | 1 | 3 |
| 4511 | conserved hypothetical protein. Uncharacterized proteins, homologs of lactam utilization protein B [General function prediction only]. | 1 | 0 | 0 | 0 | -1 | -1 |
| 4523 | hypothetical protein. Between ampD and nadC | 5 | 6 | 6 | 3 | 4 | 0 |
| 4631 | hypothetical protein. COG predictions: WcaG, Nucleoside-diphosphate-sugar epimerases [Cell envelope biogenesis, outer membrane / Carbohydrate transport and metabolism]. | 0 | 0 | -1 | 1 | -1 | -1 |
| 4632 | hypothetical protein. COG predictions: HtpX, Zn-dependent protease with chaperone function [Posttranslational modification, protein turnover, chaperones]. | -1 | -1 | 1 | 1 | -1 | -1 |
| 4686 | probable short-chain dehydrogenase. COG predictions: FabG, Dehydrogenases with different specificities (related to short-chain alcohol dehydrogenases) [Secondary metabolites biosynthesis, transport, and catabolism / General function prediction only]. | 4 | 6 | 6 | 2 | 4 | 1 |
| 4691 | hypothetical protein. COG predictions: Predicted membrane protein [Function unknown]. | 4 | 6 | 7 | 3 | 4 | 2 |
| 5184 | hypothetical protein. COG predictions: PheA, Chorismate mutase [Amino acid transport and metabolism]. | 0 | 4 | 6 | 7 | 3 | 0 |
| 5208 | conserved hypothetical protein. COG predictions: Phosphate transport regulator (distant homolog of PhoU) [Inorganic ion transport and metabolism]. | 0 | 0 | -1 | 1 | -1 | 3 |
| 5307 | hypothetical protein | 2 | 0 | 0 | 0 | -1 | -1 |
| 5433 | conserved hypothetical protein. COG predictions: RimL, Acetyltransferases, including N-acetylases of ribosomal proteins [Translation, ribosomal structure and biogenesis]. | 1 | 0 | 2 | 2 | 1 | 1 |
| 5457 | hypothetical protein. COG predictions: UbiG, 2-polyprenyl-3-methyl-5-hydroxy-6-metoxy-1,4-benzoquinol methylase [Coenzyme metabolism] | 1 | 0 | -1 | 0 | -2 | -1 |
| 5528** | hypothetical protein | 1 | -1 | -1 | 1 | 0 | 2 |
| 5540 | hypothetical protein. COG predictions: PaaY, Carbonic anhydrases/acetyltransferases, isoleucine patch superfamily [General function prediction only]. | 5 | 6 | 6 | 3 | 4 | 0 |
| 0809** | probable transporter. COG predictions: MntH, Mn2+ and Fe2+ transporters of the NRAMP family [Inorganic ion transport and metabolism]. | 6 | 6 | 6 | 2 | 4 | 1 |
| 1048** | probable outer membrane protein precursor. COG predictions: OmpA, Outer membrane protein and related peptidoglycan-associated (lipo)proteins [Cell envelope biogenesis, outer membrane]. | 2 | 6 | 6 | 3 | 4 | 1 |
| 1408** | hypothetical protein. COG predictions: Small-conductance mechanosensitive channel [Cell envelope biogenesis, outer membrane]. | 1 | 0 | 3 | 3 | 0 | 5 |
| 1436** | probable Resistance-Nodulation-Cell Division (RND) efflux transporter. COG predictions: AcrB, Cation/multidrug efflux pump [Defense mechanisms]. | 0 | 0 | -1 | 1 | -1 | -1 |
| 2006** | probable major facilitator superfamily (MFS) transporter. COG predictions: UhpC, Sugar phosphate permease [Carbohydrate transport and metabolism]. | 1 | 0 | 2 | 2 | 0 | 4 |
| 2435** | probable cation-transporting P-type ATPase . COG predictions: ZntA, Cation transport ATPase [Inorganic ion transport and metabolism]. | -1 | 0 | 1 | 0 | -1 | 2 |
| 2522** | czcC. Outer membrane protein precursor CzcC. COG predicitions: TolC, Outer membrane protein [Cell envelope biogenesis, outer membrane / Intracellular trafficking and secretion]. | 5 | 6 | 6 | 3 | 4 | 1 |
| 2527** | probable Resistance-Nodulation-Cell Division (RND) efflux transporter. Membrane proteins ; Transport of small molecules | -1 | 1 | 5 | 1 | 0 | -1 |
| 3877* | Nark1: nitrite extrusion protein 1. Membrane proteins ; Transport of small molecules | 5 | 6 | 6 | 2 | 3 | 1 |
| 3893* | conserved hypothetical protein COG predictions: Predicted membrane protein [Function unknown]. | 0 | 0 | -1 | 1 | 0 | 3 |
| 4008** | probable hydrolase. COG predictions: MhpC, Predicted hydrolases or acyltransferases (alpha/beta hydrolase superfamily) [General function prediction only]. | 0 | 0 | -1 | 1 | -1 | -1 |
| 4747** | secG . secretion protein SecG. COG predictions: Preprotein translocase subunit SecG [Intracellular trafficking and secretion]. | 4 | 6 | 6 | 2 | 4 | 2 |
| 5097** | probable amino acid permease class 3. COG predictions: AnsP, Gamma-aminobutyrate permease and related permeases [Amino acid transport and metabolism]. | 0 | 0 | 0 | 1 | -1 | 5 |
| 5528** | hypothetical protein | 1 | -1 | -1 | 1 | 0 | 2 |
| 5529** | probable sodium/proton antiporter . COG predictions: KefB, Kef-type K+ transport systems, membrane components [Inorganic ion transport and metabolism]. | 1 | 0 | 2 | 2 | 0 | 2 |
| 1727 | conserved hypothetical protein. COG predictions: Predicted signal transduction protein containing a membrane domain, an EAL and a GGDEF domain [Signal transduction mechanisms]. | 2 | 5 | 6 | 2 | 4 | 1 |
| 3704** | wspE . probable chemotaxis sensor/effector fusion protein. COG predictions: CheA, Chemotaxis protein histidine kinase and related kinases [Cell motility and secretion / Signal transduction mechanisms]. | 0 | 0 | -1 | 1 | -1 | 4 |
| 4367 | conserved hypothetical protein. Predicted signal transduction protein containing a membrane domain, an EAL and a GGDEF domain [Signal transduction mechanisms].Motility & Attachment | 3 | 5 | 6 | 2 | 4 | 0 |
| 4552 | pilW. type 4 fimbrial biogenesis protein PilW. COG predictions: PilW, Tfp pilus assembly protein PilW [Cell motility and secretion / Intracellular trafficking and secretion]. | -1 | 0 | 2 | 1 | -1 | 0 |
| 0464 | creC. two-component sensor CreC. Alternate gene name: phoM. COG predictions: BaeS, Signal transduction histidine kinase [Signal transduction mechanisms]. | -1 | 0 | 0 | 1 | -1 | 5 |
| 3704** | wspE . probable chemotaxis sensor/effector fusion protein. COG predictions: CheA, Chemotaxis protein histidine kinase and related kinases [Cell motility and secretion / Signal transduction mechanisms]. | 0 | 0 | -1 | 1 | -1 | 4 |
| 4112 | hypothetical protein. probable sensor/response regulator hybrid. Two-component regulatory systems.COG predictions: Predicted periplasmic ligand-binding sensor domain [Signal transduction mechanisms]. | 6 | 6 | 6 | 3 | 4 | 1 |
| 5484 | probable two-component sensor. COG predictions: VicK, Signal transduction histidine kinase [Signal transduction mechanisms]. | 5 | 6 | 6 | 3 | 4 | 1 |
| 0863 | probable oxidoreductase. Qor, NADPH:quinone reductase and related Zn-dependent oxidoreductases [Energy production and conversion / General function prediction only]. | 0 | 0 | 3 | 0 | -1 | 2 |
| 1821 | probable enoyl-CoA hydratase/isomerase. COG predictions: CaiD, Enoyl-CoA hydratase/carnithine racemase [Lipid metabolism]. | 1 | 3 | 3 | 2 | 1 | 2 |
| 1997 | probable AMP-binding enzyme. COG predictions: Acs, Acyl-coenzyme A synthetases/AMP-(fatty) acid ligases [Lipid metabolism]. | -1 | 0 | 1 | 1 | -1 | -3 |
| 3328 | probable FAD-dependent monooxygenase. COG predictions: UbiH, 2-polyprenyl-6-methoxyphenol hydroxylase and related FAD-dependent oxidoreductases [Coenzyme metabolism / Energy production and conversion]. | 1 | 0 | 3 | 3 | -1 | 5 |
| 3723 | Probable FMN oxidoreductase. COG predictions: NemA, NADH:flavin oxidoreductases, Old Yellow Enzyme family [Energy production and conversion]. | 2 | 6 | 6 | 3 | 4 | 1 |
| 4008** | probable hydrolase. COG predictions: MhpC, Predicted hydrolases or acyltransferases (alpha/beta hydrolase superfamily) [General function prediction only]. | 0 | 0 | -1 | 1 | -1 | -1 |
| 4131 | probable iron-sulfur protein. NapH, Polyferredoxin [Energy production and conversion]. | 6 | 6 | 6 | 2 | 4 | 0 |
| 4715 | probable aminotransferase. COG predictions: Aspartate/tyrosine/aromatic aminotransferase [Amino acid transport and metabolism]. | 2 | 0 | 0 | 0 | -1 | -1 |
| 5190 | probable nitroreductase. NfnB, Nitroreductase [Energy production and conversion]. | 1 | 1 | -1 | 0 | -1 | 0 |
| 5542 | hypothetical protein. COG predictions: AmpC, Beta-lactamase class C and other penicillin binding proteins [Defense mechanisms]. | 0 | 0 | 0 | 0 | -1 | -1 |
| 3704** | wspE . probable chemotaxis sensor/effector fusion protein. COG predictions: CheA, Chemotaxis protein histidine kinase and related kinases [Cell motility and secretion / Signal transduction mechanisms]. | 0 | 0 | -1 | 1 | -1 | 4 |
| 3096 | xcpY. general secretion pathway protein L. COG predictions: PulL, Type II secretory pathway, component PulL [Intracellular trafficking and secretion]. | -1 | 0 | 0 | 0 | -1 | 0 |
| 4747** | secG . secretion protein SecG. COG predictions: Preprotein translocase subunit SecG [Intracellular trafficking and secretion]. | 4 | 6 | 6 | 2 | 4 | 2 |
| 3319 | plcN: non-hemolytic phospholipase C precursor. Secreted Factors (toxins, enzymes, alginate) | 4 | 6 | 6 | 2 | 4 | -1 |
| 3337 | rfaD. ADP-L-glycero-D-mannoheptose 6-epimerase. COG predictions: WcaG, Nucleoside-diphosphate-sugar epimerases [Cell envelope biogenesis, outer membrane / Carbohydrate transport and metabolism]. | 7 | 6 | 6 | 2 | 4 | 1 |
| 4130 | probable sulfite or nitrite reductase COG predictions: CysI, Sulfite reductase, beta subunit (hemoprotein) [Inorganic ion transport and metabolism]. | 6 | 6 | 4 | 2 | 4 | 1 |
| 2194 | hcnB. hydrogen cyanide synthase HcnB. Class 2. | 5 | 6 | 6 | 3 | 4 | 2 |
| 4598** | mexD. Resistance-Nodulation-Cell Division (RND) multidrug efflux transporter MexD. Transport of small molecules ; Membrane proteins ; Antibiotic resistance and susceptibility | -2 | 0 | -1 | 0 | -1 | 0 |
| 1311 | phnX . 2-phosphonoacetaldehyde hydrolase. Carbon compound catabolism | 0 | 1 | 3 | 1 | -1 | 0 |
| 1931** | probable ferredoxin. COG predictions: CoxS, Aerobic-type carbon monoxide dehydrogenase, small subunit CoxS/CutS homologs [Energy production and conversion]. | 1 | 0 | 0 | 0 | -1 | -1 |
| 2265 | gluconate dehydrogenase. COG predictions: BetA, Choline dehydrogenase and related flavoproteins [Amino acid transport and metabolism]. | 4 | 6 | 6 | 3 | 4 | 0 |
| 2574 | alkB1. alkane-1-monooxygenase. Carbon compound catabolism | 1 | -1 | -1 | 0 | -1 | 5 |
| 3195 | gapA. glyceraldehyde 3-phosphate dehydrogenase | 2 | 6 | 6 | 2 | 4 | 1 |
| 3570** | mmsA. methylmalonate-semialdehyde dehydrogenase | 6 | 6 | 6 | 3 | 3 | 1 |
| 5349 | probable rubredoxin reductase. COG predictions: NirB, NAD(P)H-nitrite reductase [Energy production and conversion]. | 0 | 0 | -1 | 1 | -1 | 0 |
| 0849/2616 | PA0849: thioredoxin reductase 2. COG predictions: TrxB, Thioredoxin reductase [Posttranslational modification, protein turnover, chaperones].  PA2616: thioredoxin reductase 1. COG predictions: TrxB, Thioredoxin reductase [Posttranslational modification, protein turnover, chaperones]. | 0 | 0 | 3 | 1 | -1 | -1 |
| 0997 | pqsB . Homologous to beta-keto-acyl-acyl-carrier protein synthase. Biosynthesis of cofactors, prosthetic groups and carriers | 0 | 0 | -1 | 1 | 0 | 3 |
| 4919 | pncB1. nicotinate phosphoribosyltransferase. | 0 | 2 | 3 | 3 | -1 | 5 |
| 1931** | probable ferredoxin. COG predictions: CoxS, Aerobic-type carbon monoxide dehydrogenase, small subunit CoxS/CutS homologs [Energy production and conversion]. | 1 | 0 | 0 | 0 | -1 | -1 |
| 2664 | fhp.flavohemoprotein. COG predictions: Hmp, Hemoglobin-like flavoprotein [Energy production and conversion]. | 1 | 6 | 6 | 2 | 4 | 0 |
| 3195 | gapA. glyceraldehyde 3-phosphate dehydrogenase | 2 | 6 | 6 | 2 | 4 | 1 |
| 3924 | probable medium-chain acyl-CoA ligase. COG predicitons: CaiC, Acyl-CoA synthetases (AMP-forming)/AMP-acid ligases II [Lipid metabolism / Secondary metabolites biosynthesis, transport, and catabolism]. | 0 | -1 | 3 | 3 | 0 | 4 |
| 4234 | uvrA . excinuclease ABC subunit A. DNA replication, recombination, modification and repair. | 1 | 1 | 3 | 1 | -1 | 5 |
| 4468 | sodA. superoxide dismutase | 0 | 0 | 3 | 2 | -1 | 2 |
| 5372** | betA . choline dehydrogenase. Amino acid biosynthesis and metabolism ; Adaptation, Protection | -2 | -1 | 0 | 1 | 0 | 2 |
| 5474 | probable metalloprotease. COG predicitions: PqqL, Predicted Zn-dependent peptidases [General function prediction only]. | 1 | 0 | 3 | 3 | 0 | 2 |
| 0619 | probable bacteriophage protein. COG predictions: gpI, Bacteriophage P2-related tail formation protein [General function prediction only]. | 0 | 0 | 0 | 1 | 0 | -1 |
| 3434; 2319; 4797; 3993; 0445; 2690 | probable transposase. Related to phage, transposon, or plasmad. COG predictions: Transposase and inactivated derivatives [DNA replication, recombination, and repair]. | 0 | 0 | 2 | 2 | 1 | 2 |
| 1738 | Probable transcriptional regulator. COG prediction: LysR, Transcriptional regulator [Transcription]. | 3 | 6 | 6 | 2 | 4 | 0 |
| 1759 | probable transcriptional regulator. MalT, ATP-dependent transcriptional regulator [Transcription]. | 1 | 0 | 3 | 2 | 0 | 3 |
| 2591 | probable transcriptional regulator. COG predictions: CitB, Response regulator containing a CheY-like receiver domain and an HTH DNA-binding domain [Signal transduction mechanisms / Transcription]. | 0 | 7 | 7 | 2 | 5 | 5 |
| 4787 | probable transcriptional regulator. COG predictions: AraC, AraC-type DNA-binding domain-containing proteins [Transcription]. | 4 | 6 | 6 | 3 | 4 | 3 |
| 5380 | probable transcriptional regulator. COG predictions: Transcriptional regulator containing an amidase domain and an AraC-type DNA-binding HTH domain [Transcription]. | 1 | 0 | 0 | 1 | 0 | 0 |
| 0809** | probable transporter. COG predictions: MntH, Mn2+ and Fe2+ transporters of the NRAMP family [Inorganic ion transport and metabolism]. | 6 | 6 | 6 | 2 | 4 | 1 |
| 0958 | oprD. Basic amino acid, basic peptide and imipenem outer membrane porin OprD precursor | 0 | 0 | 3 | 1 | 3 | 0 |
| 1048** | probable outer membrane protein precursor. COG predictions: OmpA, Outer membrane protein and related peptidoglycan-associated (lipo)proteins [Cell envelope biogenesis, outer membrane]. | 2 | 6 | 6 | 3 | 4 | 1 |
| 1410 | probable periplasmic spermidine/putrescine-binding protein class 3: COG predictions: PotD, Spermidine/putrescine-binding periplasmic protein [Amino acid transport and metabolism]. | 5 | 6 | 6 | 3 | 4 | 1 |
| 1436** | probable Resistance-Nodulation-Cell Division (RND) efflux transporter. COG predictions: AcrB, Cation/multidrug efflux pump [Defense mechanisms]. | 0 | 0 | -1 | 1 | -1 | -1 |
| 2006** | probable major facilitator superfamily (MFS) transporter. COG predictions: UhpC, Sugar phosphate permease [Carbohydrate transport and metabolism]. | 1 | 0 | 2 | 2 | 0 | 4 |
| 2435** | probable cation-transporting P-type ATPase . COG predictions: ZntA, Cation transport ATPase [Inorganic ion transport and metabolism]. | -1 | 0 | 1 | 0 | -1 | 2 |
| 2522** | czcC. Outer membrane protein precursor CzcC. COG predicitions: TolC, Outer membrane protein [Cell envelope biogenesis, outer membrane / Intracellular trafficking and secretion]. | 5 | 6 | 6 | 3 | 4 | 1 |
| 2527** | probable Resistance-Nodulation-Cell Division (RND) efflux transporter. Membrane proteins ; Transport of small molecules | -1 | 1 | 5 | 1 | 0 | -1 |
| 2760 | probable outer membrane protein precursor. Transport of small molecules. | 1 | 1 | 4 | 4 | 1 | 4 |
| 3877** | Nark1: nitrite extrusion protein 1. Membrane proteins ; Transport of small molecules | 5 | 6 | 6 | 2 | 3 | 1 |
| 4598** | mexD. Resistance-Nodulation-Cell Division (RND) multidrug efflux transporter MexD. Transport of small molecules ; Membrane proteins ; Antibiotic resistance and susceptibility | -2 | 0 | -1 | 0 | -1 | 0 |
| 4913 | probable binding protein component of ABC transporter. COG predictions: LivK, ABC-type branched-chain amino acid transport systems, periplasmic component [Amino acid transport and metabolism]. | 1 | 0 | 3 | 3 | 0 | 4 |
| 5097** | probable amino acid permease class 3. COG predictions: AnsP, Gamma-aminobutyrate permease and related permeases [Amino acid transport and metabolism]. | 0 | 0 | 0 | 1 | -1 | 5 |
| 5529** | probable sodium/proton antiporter . COG predictions: KefB, Kef-type K+ transport systems, membrane components [Inorganic ion transport and metabolism]. | 1 | 0 | 2 | 2 | 0 | 2 |
| 1326 and 0331 | PA1326: ilvA2. threonine dehydratase, biosynthetic. Amino acid biosynthesis and metabolism.  PA0331: ilvA1. threonine dehydratase, biosynthetic. Amino acid biosynthesis and metabolism | 0 | 1 | 2 | 2 | 1 | 1 |
| 3570** | mmsA. methylmalonate-semialdehyde dehydrogenase | 6 | 6 | 6 | 3 | 3 | 1 |
| 5263 | argH. argininosuccinate lyase. Amino acid biosynthesis and metabolism | 4 | 6 | 6 | 3 | 4 | 0 |
| 5372** | betA . choline dehydrogenase. Amino acid biosynthesis and metabolism ; Adaptation, Protection | -2 | -1 | 0 | 1 | 0 | 2 |
| Between 0599 and 0600 | Intergenic region | 6 | 6 | 6 | 2 | 4 | 0 |
| Between 2460 and 2461 | Intergenic region | 0 | 1 | 0 | 2 | 0 | 2 |
| Between 2751 and 2752 | Intergenic region | 0 | 0 | -1 | 1 | 1 | -1 |
| Between 3450 and 3451 | Intergenic region | 1 | 0 | 2 | 2 | 0 | 5 |
| Between 4654 and 4655 | Intergenic region | 2 | 6 | 3 | 0 | 4 | 0 |
| Between 4805 and 4806 | Intergenic region | 5 | 6 | 6 | 2 | 4 | 0 |
| Between 5410 and 5411 | Intergenic region | 0 | 0 | 2 | 2 | 3 | 3 |
| PFL2789 | pltC. polyketide synthase. Cellular processes: Toxin production and resistance | 1 | 2 | 0 | 0 | -1 | 3 |
| STM2714 | Fels-2 prophage: similar to lysis protein (lysB). Salmonella typhimurium | 1 | 1 | 2 | 1 | 1 | 4 |
| XCC2973 | phage-related terminase. X.campestris | 0 | 0 | 0 | 4 | 0 | 3 |
| Rsc1931  Bpsl0163  Xoo1710 | Putative phage related protein (hydrolase). Ralstonia solacenarum. TIGR Cellular Role Category: Cell envelope: Biosynthesis and degradation of murein sacculus and peptidoglycan | -1 | 0 | 0 | 1 | 0 | 2 |
| PSPPH_1926 | pyoverdine sidechain peptide synthetase IV, D-Asp-L-Ser component. Cellular processes: Pathogenesis, Biosynthesis of natural products, Biosynthesis of cofactors, prosthetic groups, and carriers: Siderophores | -2 | -1 | -1 | 1 | 2 | -1 |

***:** Relative antibiotic susceptibility was expressed as log_2_(MIC mutant)/(MIC wild-type), where MIC is the minimal inhibitory concentration for a given antibiotic.

****:** Appears in two or more classes

Pol, polymixin B; Amk, amikacin; Cip, ciprofloxacin; Tet, tetracycline; Imi, imipenem; Cef, ceftazidime.

|  | **HYPOTHETICAL, UNCLASSIFIED, UNKNOWN** |
| --- | --- |
|  | **MEMBRANE PROTEINS** |
|  | **MOTILITY AND ATTACHMENT** |
|  | **TWO COMPONENT REGULATORY SYSTEMS** |
|  | **PUTATIVE ENZYMES** |
|  | **CHEMOTAXIS** |
|  | **PROTEIN SECRETION/EXPORT APPARATUS** |
|  | **SECRETED FACTORS: TOXINS, ENZYMES, ALGINATE** |
|  | **CELL WALL/LPS/CAPSULE** |
|  | **CENTRAL INTERMEDIARY METABOLISM** |
|  | **ANTIBIOTIC RESISTANCE AND SUSCEPTIBILITY** |
|  | **CARBON COMPOUND CATABOLISM** |
|  | **NUCLEOTIDE BIOSYNTHESIS AND METABOLISM** |
|  | **BIOSYNTHESIS OF COFACTORS, PROSTHETIC GROUPS AND CARRIERS** |
|  | **ENERGY METABOLISM** |
|  | **FATTY ACID AND PHOSPHOLIPID METABOLISM** |
|  | **DNA REPLICATION, RECOMBINATION, MODIFICATION AND REPAIR** |
|  | **ADAPTATION, PROTECTION** |
|  | **TRANSLATION, POST-TRANSLATION MODIFICATION, DEGRADATION** |
|  | **RELATED TO PHAGE, TRANSPOSON OR PLASMID** |
|  | **TRANSCRIPTIONAL REGULATORS** |
|  | **TRANSPORT OF SMALL MOLECULES** |
|  | **AMINOACID BIOSYNTHESIS AND METABOLISM** |
|  | **INTERGENIC REGIONS** |
|  | **NON-PAO1 ORFs: The name corresponds to the most homologous gene present in databases** |
